# Discovery and Identification of Pyrazolopyramidine Analogs as Novel Potent Androgen Receptor Antagonists

**DOI:** 10.3389/fphar.2018.00864

**Published:** 2018-08-28

**Authors:** Lingyan Wang, Tianqing Song, Xin Wang, Jiazhong Li

**Affiliations:** School of Pharmacy, Lanzhou University, Lanzhou, China

**Keywords:** prostate cancer, androgen receptor, virtual screening, pyrazolopyrimidine, AR reporter gene assay, molecular dynamics

## Abstract

Androgen receptor (AR), an important target in the current androgen derivation therapy, plays a critical role in the development and progress of prostate cancer (PCa). Nonsteroidal antiandrogens, such as enzalutamide and bicalutamide, are commonly used in clinic to treat PCa. Though they are very effective at the beginning, drug resistance problem appears after about 18 months. One of the reasons is that these antiandrogens share similar structure skeleton. Therefore, it is urgent to discover novel antiandrogens with different skeletons for resistance problem. Herein, we combined structure- and ligand-based methodologies for virtual screening chemical databases to identify potent AR antagonists. Then the cytotoxic activities of the screened hit samples were evaluated by using LNCaP prostate cancer cells. Virtual screening and biological evaluation assay results suggest that several chemicals with novel pyrazolopyrimidine skeleton can inhibit the proliferation of prostate cancer cells with similar, or even higher, bioactivities to bicalutamide. AR reporter gene assay experiments proved that Compound III showed potential antagonistic effects. In addition, molecular dynamics simulations results proved that Compound III can properly bind to AR and prevent helix 12 (H12) from closing to distort the formation of activation function 2 (AF2) site, resulting in the invalid transcription. Hence, pyrazolopyrimidine was discovered as a novel, potent and promising antiandrogen skeleton deserved to be further studied.

## Introduction

According to the latest cancer statistics 2016 (Siegel et al., 2016), prostate cancer (PCa) is the second most common cancer among males around the world. The estimated death rate of PCa is 8%, which is just lower than the leading lung cancer, but the estimated new cases of prostate cancer become highest among the diagnosed cancers. Androgen receptor (AR) plays a critical role not only in the development of prostate cancer but also in the progress of the advanced castration states (Tilley et al., 1994; Taplin et al., 2003; Jernberg et al., 2017). Therefore, effective suppression of AR activity remains mainstream therapeutic schemes to the treatment of advanced, recurrent and metastatic prostate cancer.

AR, a class of ligand-activated transcription factor, is a member of the steroid and nuclear receptor (NR) superfamily (Evans, 1988). The AR structure consists of four basic elements: N-terminal domain (NTD), DNA-binding domain (DBD), hinge region and ligand-binding domain (LBD), that is a highly structurally conserved throughout the NR superfamily (Gao et al., 2005). Natural hormone testosterone (T) and dihydrotestosterone (DHT), binding to AR LBD, are the endogenous ligands of AR. The main mechanism of androgen action is to regulate the gene expression, change the level of specific proteins in cells and control cells behavior (Fix et al., 2004). Once bound to AR, androgens play pivotal roles in the sexual development, function, musculoskeletal growth of male and the progress of prostate cancer.

The standard treatment of prostate cancer involves androgen derivation therapy in conjunction with small molecule antiandrogens that block AR signaling (Hodgson et al., 2007). Antiandrogens compete with DHT for the binding to AR, inhibiting AR transactivation through a variety of mechanisms, including disruption of nuclear localization, interruption of DNA binding and interference with co-activator recruitment (Tran et al., 2009; Sadar, 2012). Unfortunately, most patients receiving antiandrogen therapy eventually develop drug resistance problem indicated by the rising level of serum prostate-specific antigen (PSA), leading to the lethal disease state termed castration-resistant prostate cancer (CRPC) (Denmeade et al., 2003). To deal with this situation, more efforts have been concentrated on design and discovery of new AR antagonists with novel skeleton to overcome drug resistance in CRPC.

In the current study, a combined structure- and ligand-based screening strategy was applied to virtually screen a big commercial chemical database to fish potential AR antagonists. Considering the published AR protein crystal structures are all in the agonistic manner (Bohl et al., 2005a,b), we firstly constructed its antagonistic conformation by using homology modeling according to the reported process (Liu et al., 2012). Structure-based docking method was used to find out chemicals that could bind to AR with high affinities. Then, a set of strictly validated QSAR models were used to predict the *in silico* antiandrogen abilities of the screened chemicals. Subsequently, the cell proliferation and AR reporter gene assay were used to evaluate the biological activities of the filtered molecules. Finally, molecular dynamics (MD) simulations and molecular mechanics Generalized Born (GB) surface area (MM-GBSA) calculations were applied to explore the interaction mechanics between pyrazolopyrimidine and ARs including wild type (WT), F876L and H874Y mutant types.

## Results and discussion

**Figure 1:**
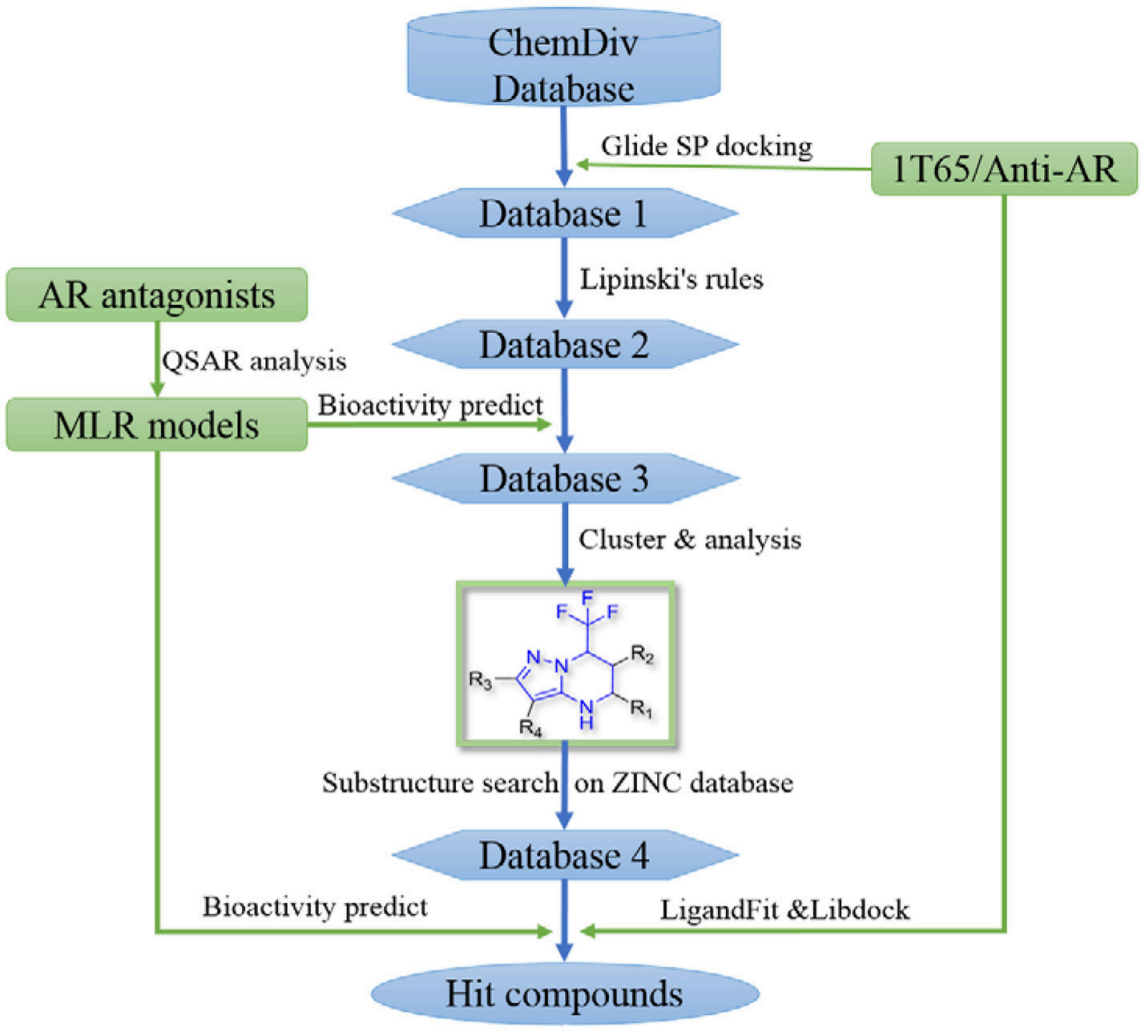
The flowchart of the combination of structure- and ligand- based virtual screening strategy.

In order to effectively dig out chemicals with high antiandrogen potency, a combined structure- and ligand-based strategy was applied in this work. The flowchart of the combined virtual screening procedure was shown in Figure 1.

### Structure-based virtual screening

The AR ligand binding domain represents a hydrophobic pocket, which can accommodate various chemicals of different sizes and chemotypes. Taking into account that the crystal structure of AR with antagonistic manner (Anti-AR) is unclear, homology modeling was applied to construct its Anti-AR conformation using the same method as previously reported (Liu et al., 2012). Then the AR crystal structure (PDB ID:1T65) and Anti-AR were both used to virtually screen the ChemDiv database by using Glide standard precision (SP) docking program. Glide SP, a semi flexible docking method, performs exhaustive sampling and is the recommended balance between speed and accuracy.

**Figure 2:**
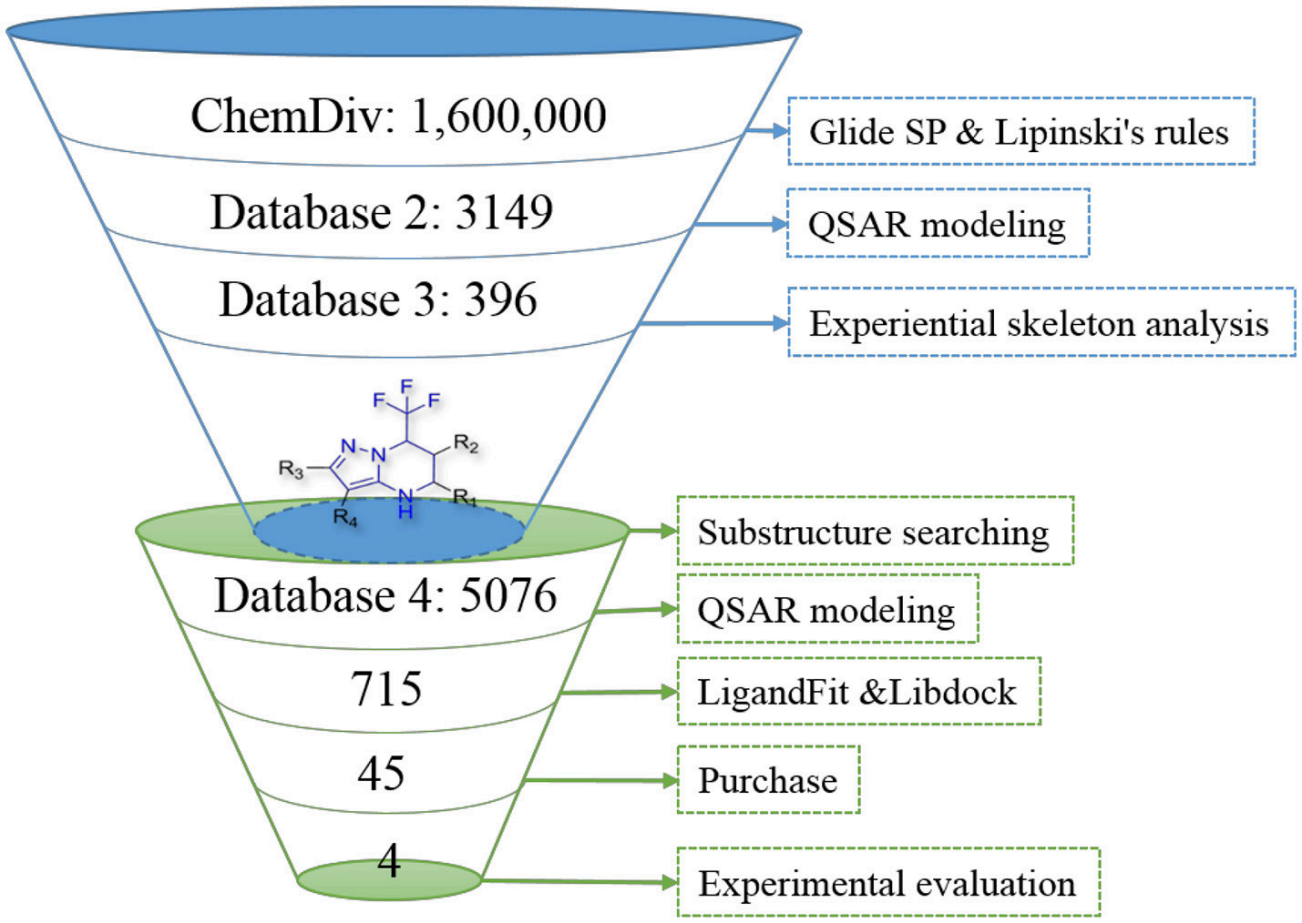
The results of screening process step by step.

ChemDiv is now the recognized global leader in discovery chemistry with the industry's largest, most diverse, and most pharmacologically-relevant commercial collection of 1.6 million individually crafted, lead-like, drug-like small molecules etc. All chemicals that can be docked into the ligand binding domains of both 1T65 and Anti-AR were reserved. Subsequently, these compounds were further filtered following Lipinski's rules of five, including less than five hydrogen-bond donors, ten hydrogen-bond acceptors, 500 Da of molecular weight and AlogP of 5 in Discovery Studio 2.5, to keep compounds with potential bioavailability. Finally, 3,149 compounds were reserved in Database 2 for further screening, as shown in Figure 2.

### Ligand-based virtual screening

Quantitative structure-activity relationship (QSAR) is an analytical methodology that can be used to interpret the quantitative relationship between molecular structures and corresponding biological activities. Most importantly, after thorough validation, QSAR models can be used to quantitatively predict the activities of new chemicals. In this study, four QSAR models constructed by using multiple linear regression (MLR) method were employed to *in silico* evaluate the bioactivities of the filtered ChemDiv chemicals contained in database 2.

#### The development and validation of QSAR models

The linear MLR model Y_1_ was previously reported from our group (Wang et al., 2015). Other three models Y_2_, Y_3_, and Y_4_ were newly built. Experimental and predicted antiandrogenic activities in these three models are listed in Supporting Information Tables S1–S3. Here the antagonistic activities (IC_50_) were experimentally tested from the AR reporter gene assays. The Y in these QSAR models stands for the negative logarithmic unit of the biological activities, pIC_50_. These four linear equations were list as follows. The corresponding parameters, demonstrating the model performances, were listed in Table 1. The descriptors used to build the four linear model and corresponding meanings were listed in Table 2.

Y_1_ = 1.188GATS7v − 1.108BEHp7 − 7.281E2u + 1.453HATS4u − 2.647H6m + 28.810R6u+ − 46.815R8u+ + 12.157
Y_2_ = −2.89IC5 + 1.01GATS5e − 3.17DISPp − 12.99HATS3u + 27.13
Y_3_ = 5.601IVDE − 1.70C-009 + 1.11BLTF96 + 3.02
Y_4_ = 3.18 IC1 − 0.46F05[N-F] + 1.47R4u + 0.409 Depressant-80 + 3.85HATS7m − 7.15

**Table 1:** The QSAR MLR models and corresponding statistical parameters.

**Parameters**	**Model Y_1_**	**Model Y_2_**	**Model Y_3_**	**Model Y_4_**
R^2^	0.77	0.76	0.89	0.84
${\text{Q}}_{Loo}^{2}$	0.69	0.66	0.85	0.75
${\text{Q}}_{F1}^{2}$	0.78	0.74	0.86	0.89
${\text{Q}}_{F2}^{2}$	0.78	0.73	0.86	0.89
${\text{Q}}_{F3}^{2}$	0.85	0.88	0.86	0.76
*CCC*	0.90	0.89	0.91	0.93
${\text{RMSE}}_{\text{tr}}$^a^	0.300	0.226	0.370	0.247
${\text{RMSE}}_{\text{pred}}$^b^	0.243	0.270	0.420	0.301

a *RMSE value for the training set*.

b *bRMSE value for the prediction set*.

**Table 2:** The descriptors used to build the four linear model and corresponding meanings.

**Descriptor**	**Meaning**	**Descriptor type**
GATS7v	Geary autocorrelation - lag 7/weighted by atomic van der Waals volumes	2D autocorrelations
BEHp7	Highest eigenvalue n. 7 of Burden matrix weighted by atomic polarizabilities	Burden eigenvalues
E2u	2nd component accessibility directional WHIM index/unweighted	WHIM descriptors
HATS4u	Leverage-weighted autocorrelation of lag 4/unweighted	GETAWAY descriptors
H6m	H autocorrelation of lag 6/weighted by atomic masses	GETAWAY descriptors
R6u+	R maximal autocorrelation of lag 6/unweighted	GETAWAY descriptors
R8u+	R maximal autocorrelation of lag 8/unweighted	GETAWAY descriptors
IC5	Information content index (neighborhood symmetry of 5-order)	Information indices
GATS5e	Geary autocorrelation - lag 5/weighted by atomic Sanderson electronegativities	2D autocorrelations
DISPp	D COMMA2 value/weighted by atomic polarizabilities	Geometrical descriptors
HATS3u	Leverage-weighted autocorrelation of lag 3/unweighted	GETAWAY descriptors
IVDE	Mean information content on the vertex degree equality	Information indices
C-009	CHRX2	Atom-centered fragments
BLTF96	Verhaar model of Fish base-line toxicity from MLOGP (mmol/l)	Molecualr properties
IC1	Information content index (neighborhood symmetry of 1-order)	Information indices
F05[N-F]	Frequency of N - F at topological distance 05	2D frequency fingerprints
R4u	R autocorrelation of lag 4/unweighted	GETAWAY descriptors
Depressant-80	Ghose-Viswanadhan-Wendoloski antidepressant-like index at 80%	Molecular properties
HATS7m	Leverage-weighted autocorrelation of lag 7/weighted by atomic masses	GETAWAY descriptors

In Table 1, the parameter R^2^ describes the fitting ability and $Q_{LOO}^{2}$ represents the reliability and stability of a QSAR model. Leave-one-out cross-validated correlation coefficient ($Q_{LOO}^{2}$) is the one of internal validation methods which can avoid the risk of overfitting and the possibility of overestimating predictability. From the above parameters listed in Table 1, it can be seen that the fitting abilities of these MLR models are all high enough with *R*^2^ greater than 0.75. The predictive abilities were evaluated by means of four external validation parameters (Gramatica and Sangion, 2016), $Q_{F1}^{2}$ (www.oecd.org/dataoecd/33/37/37849783.pdf), $Q_{F2}^{2}$ (Schüürmann et al., 2008), $Q_{F3}^{3}$ (Consonni et al., 2009) and *CCC* (Lin, 1989; Chirico and Gramatica, 2011, 2012), and the formulas of parameters were shown in the Table S4. All these parameters are high enough to guarantee the predictive ability of these models. Especially the parameter *CCC* values are all beyond 0.85 as suggested in the literature (Chirico and Gramatica, 2012), which is a highly reliable parameter to guarantee the external predictability of a model. All the root mean square error (RMSE) values for the training set and prediction set are similarly low in each model. After thorough and rigorous validation, all these four QSAR models were proved to be reliable, stable and predictive and can be used to predict the bioactivities of new chemicals.

#### QSAR-based virtual screening

After models construction and validation, these four models were used to calculate the *in silico* antiandrogenic bioactivities of the filtered compounds in database 2. Firstly, these 3,149 molecules were imported to DRAGON (DRAGON for Windows, version 5.5)^1^ program to calculate all molecular descriptors. Then the descriptors involved in these four QSAR models were extracted and submitted to the equations Y_1_-Y_4_ to calculate the biological activity of each molecule. Subsequently, all these chemicals were ranked by the calculated biological activities, considering simultaneously the results from four models. Only molecules with high potency, predicted bioactivities from four models greater than 7, can be retained for the succeeding process. Finally, 396 compounds were reserved in database 3 for further structural analysis and experiential analysis.

**Figure 3:**
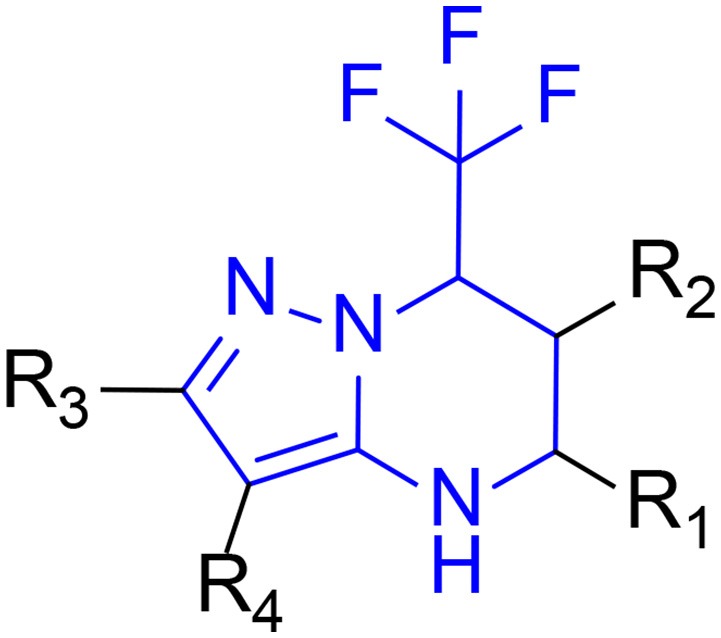
The skeleton structure of pyrazolopyrimidine analogs.

The 396 different compounds can be divided into 8 groups according to their skeleton structures. But some of them contain poisonous substructures from the point view of drug, some of them are easy to be hydrolyzed, or some of them have too complex structures etc. Finally, we focused on pyrazolopyrimidine skeleton (Figure 3) showing high *in silico* for antiandrogenic potency further study. The representative pyrazolopyrimidine analogs and corresponding predicted activities were listed in the Table 3.

**Table 3:** The details of docking score and calculated bioactivities using four models of representative pyrazolopyrimidine analogs.

**MOL_ID**	**Y_1_**	**Y_2_**	**Y_3_**	**Y_4_**	**Docking score**	**Structure**
MOL_133	7.271	10.578	9.838	7.262	−10.089	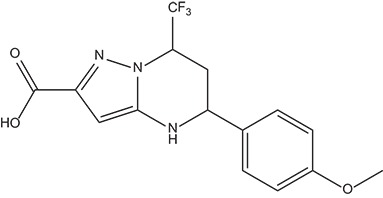
MOL_135	7.764	8.300	8.371	8.826	−9.590	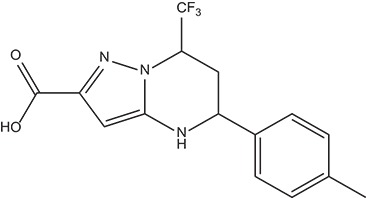

### Screening of pyrazolopyrimidine analogs

To find out more molecules with pyrazolopyrimidine skeleton, we searched the ZINC database, a free database of commercially-available compounds for virtual screening. On the ZINC website, the user can choose to search the database according to the structure similarity or substructure. In this study, our goal is to find out more molecules with pyrazolopyrimidine skeleton, so we used pyrazolopyrimidine as a substructure query to search ZINC database. Totally 5076 samples were found and downloaded to build a new chemical Database 4.

Then the four QSAR models were used again to calculate the *in silico* antiandrogenic potency of the chemicals contained in Database 4. We assumed that the chemicals with predictive value less than 7 were less bioactive, which were removed in the following study. As a result, 715 chemicals were remained for the following screening process. To find out compounds having high binding affinities with AR, the remained molecules were then imported to LigandFit and Libdock modules to do docking simulations. Taking into sufficient consideration of all the above results, we selected 45 molecules with high potency and submitted them to J&K Chemicals and SPECS. Finally we successfully purchased four compounds (Table 4), and the predicted biological activity from the Y1-Y4 models and the docking affinities were listed in Table S5.

**Table 4:** Structures and experimental activities of identified active compounds.

**No**	**CAS**	**Structure**	**IC_50_ (μM)**
Compound I	436088-54-9	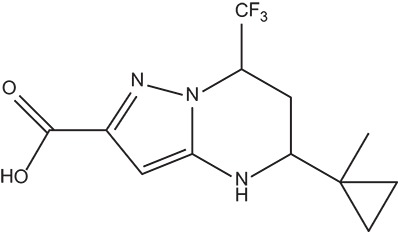	214
Compound II	312699-22-2	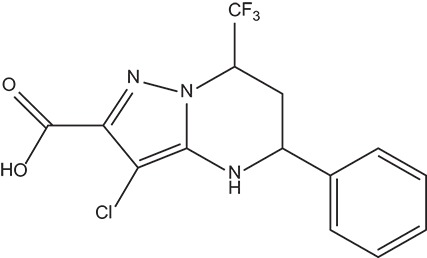	487
Compound III	332859-05-9	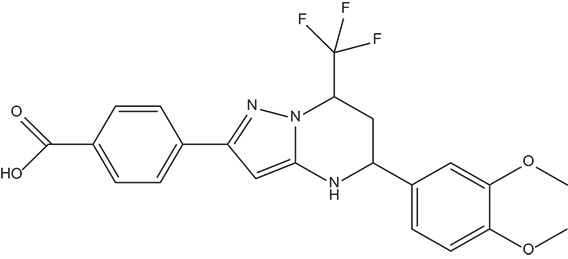	23.4 ± 4.0
Compound IV	1502817-77-7	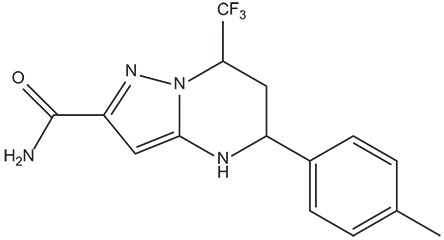	45.8 ± 2.3
R-Bicalutamide	113299-40-4	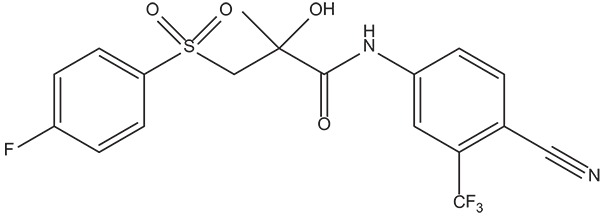	24.6 ± 4.5

### *In vitro* evaluation of hit compounds

**Figure 4:**
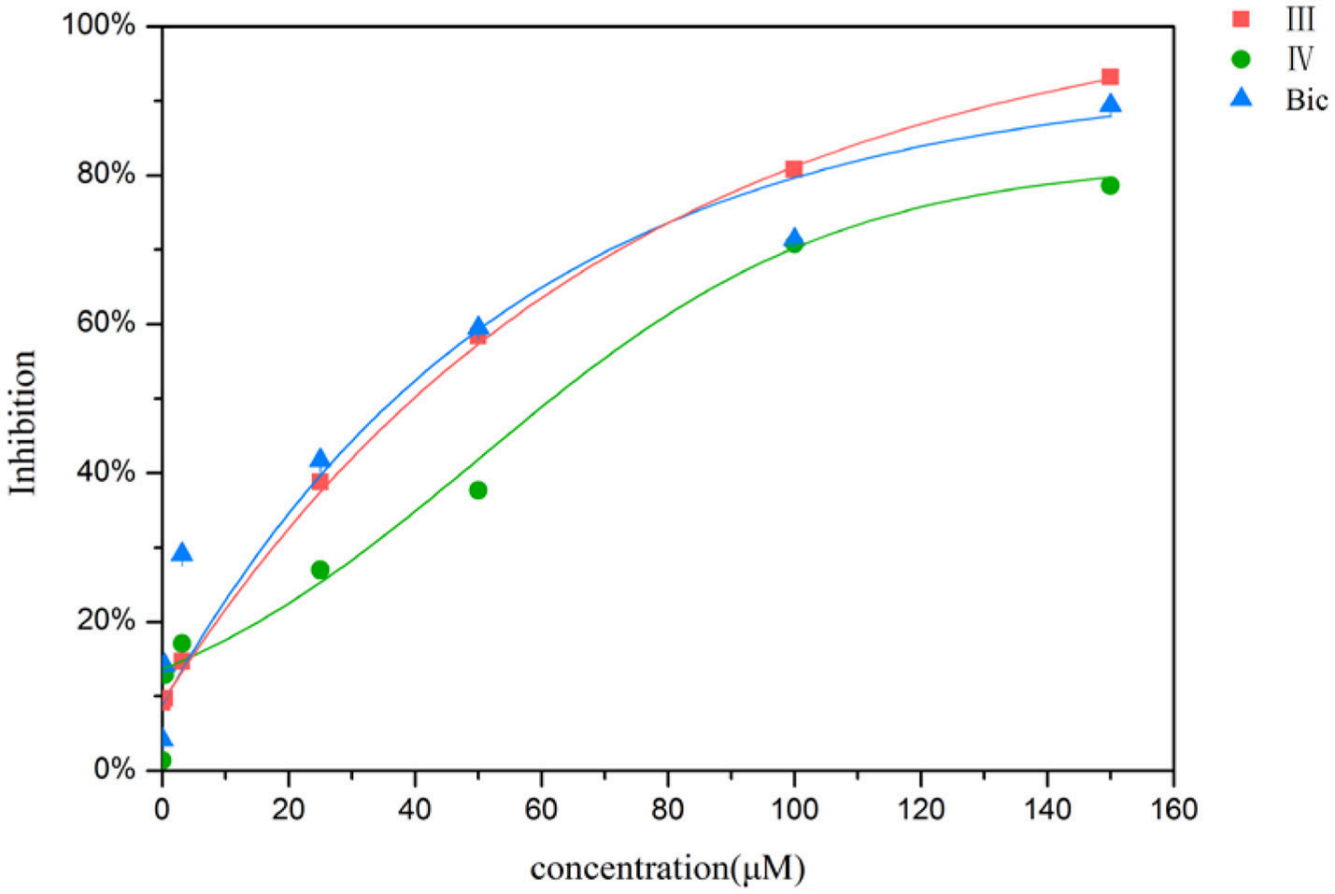
Inhibition of the proliferation for LNCaP cells by compound III, IV and bicalutamide.

In order to determine whether the screened hit compounds can affect the proliferation of prostate cancer cell, we carried out the cell proliferation assay by using the LNCaP cell line. The corresponding structures and biological activities (IC_50_) were listed in Table 4. It can be seen that all these compounds can suppress the proliferation of the LNCaP cells to varying degree, which suggests that pyrazolopyrimidine analogs can act as potential hits against CRPC. Compound IV, with the IC_50_ value of 45.8 μM, is 2-fold less active than bicalutamide. Compound III were demonstrated to be more effective than the first-generation AR antagonist bicalutamide and exhibited a dose dependent manner as shown in Figure 4.

**Figure 5:**
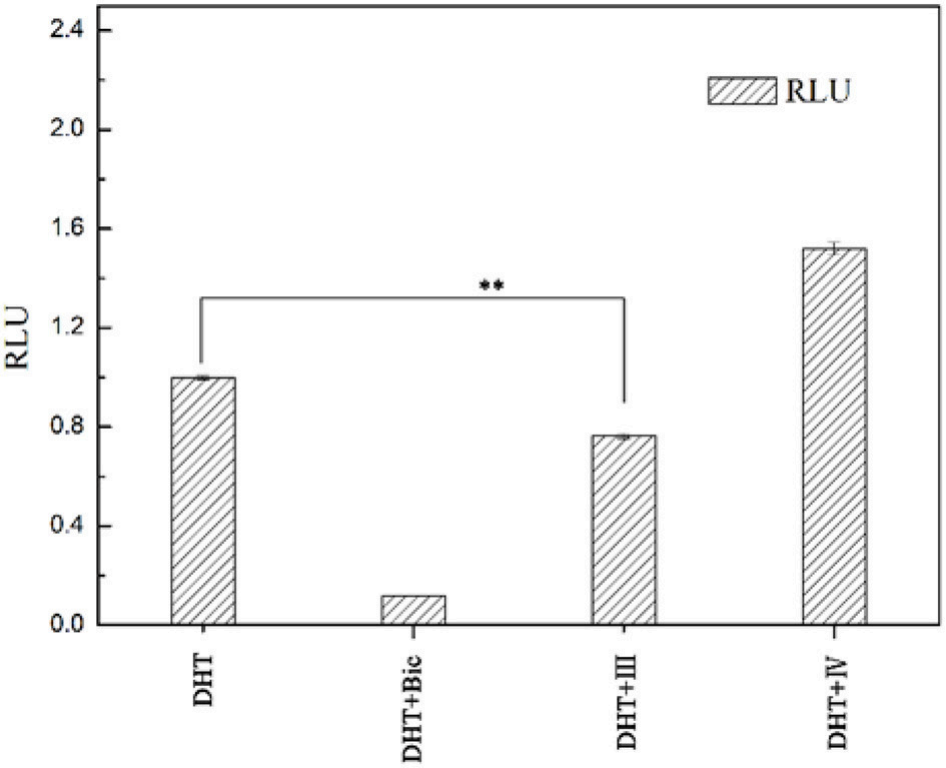
AR antagonistic activity. The COS-7 cells were treated with positive compound Bic, compound III and IV (each of 10 μM) in the presence of 10 nM DHT. **p < 0.01 compared to cells treated only with DHT. All experiments were repeated at least three times.

To examine whether these two compounds have antagonistic activities toward AR, we performed transient transfection assay with androgen reporter pMMTV-Luc, which contains the natural AR target promoters, mouse mammary tumor virus (MMTV) long terminal repeat promoters, and luciferase gene bound at the downstream of an AR promoter. The concentration of DHT is 10 nM in all AR luciferase assays. As the result, compound IV have no significantly antagonistic activity against AR, while compound III can moderately inhibit the DHT-induced transcriptional activation of AR at the concentration of 10 μM (29.8%) (Figure 5). We assumed that pyrazolopyrimidine analogs with bulky groups may play a vital role in pushing away H12 to form open conformation (antagonistic form) and hence show the antagonistic bioactivity to AR, which could be further proved by molecular dynamics simulations.

### Molecular dynamics simulation

**Figure 6:**
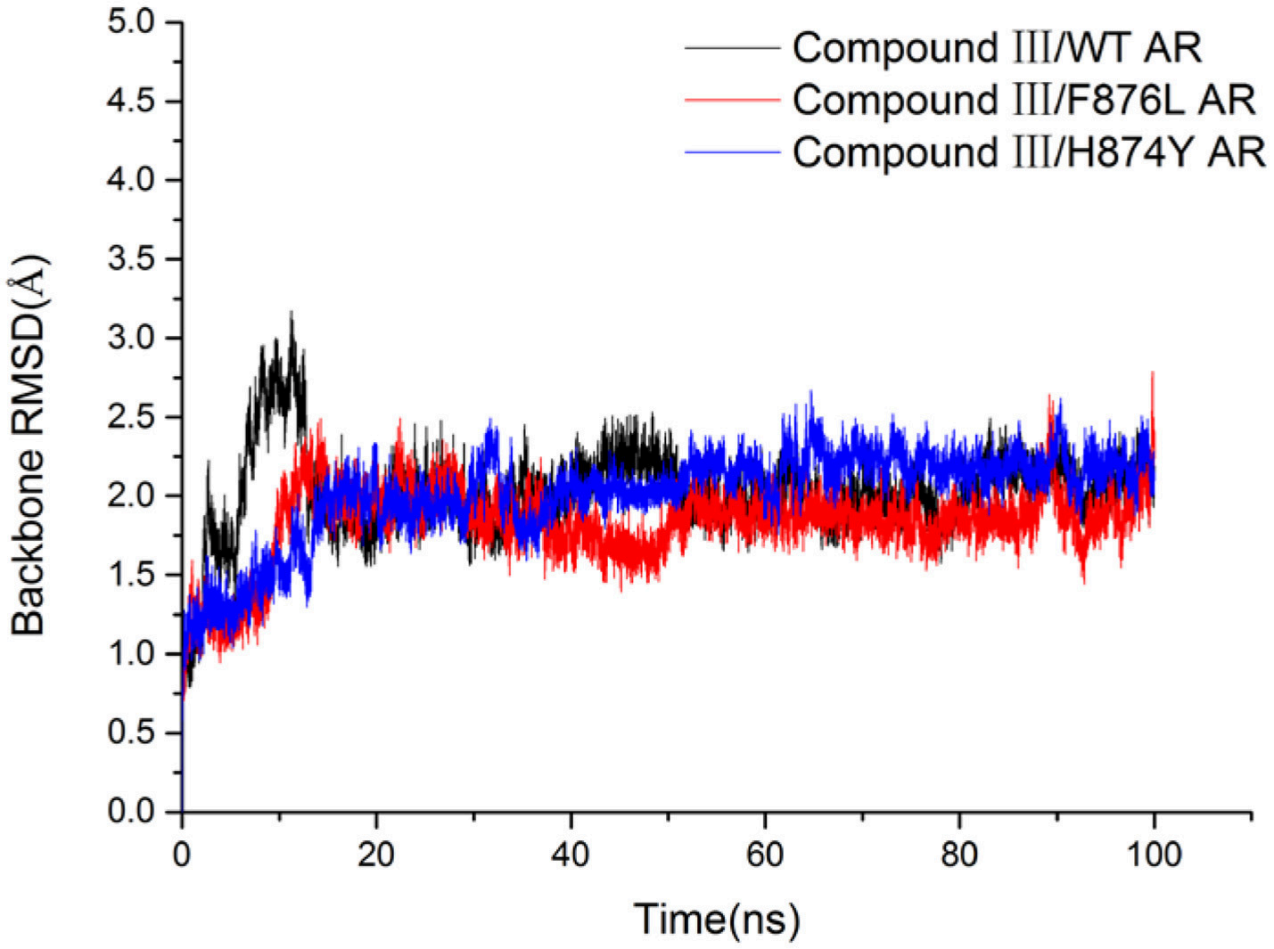
The RMSDs of backbone atoms of the AR in all three trajectories.

In order to investigate how Compound III produces antagonistic effect on androgen receptor, molecular dynamics simulations and MM/GBSA methods were employed to study the interaction between Compound III and AR, including wild type AR, F876L and H874Y mutant types, which are commonly occurred in the AR LBD and often caused enzalutamide resistance problem (Liu et al., 2017). Then free energy calculation and decomposition analysis were carried out to analyze the MD results. Figure 6 shows the root mean square deviations (RMSDs) of all the backbone atoms of the protein during all 100 ns MD, from where it can be seen that after 80 ns, the RMSD values of the protein backbone atoms and binding pocket atoms have small fluctuations, so the last 20 ns trajectories were used to do all the analysis. We analyzed the results with earlier work aimed to enzalutamide and AR (Liu et al., 2017).

In Table 5, the binding free energies of Compound III/WT AR, Compound III/F876L AR, and Compound III/H874Y AR systems, are −44.37 kcal/mol, −57.04 kcal/mol and −42.02 kcal/mol, close to the enzalutamide' s data (Liu et al., 2017). It can be seen from Table 5 that van der Waal provides the main driving force for the binding between Compound III and WT/mutant ARs. Electrostatic energies are also very important for binding except Compound III/F876L AR complex. However, the polar solvation energies are unfavorable to the binding free energy.

**Table 5:** The calculated binding free energies (kcal/mol) of the three systems by MM/GBSA method.

**Energies (kcal/mol)**	**Compound III/WT AR**	**Compound III/F876L AR**	**Compound III/H874Y AR**
ΔEele	−30.95	−100.13	8.64
ΔEvdw	−56.36	−56.08	−57.34
ΔGnp	−7.65	−7.86	−7.64
ΔGp	50.59	107.03	14.32
ΔGbind	−44.37	−57.04	−42.02

*The binding free energy (ΔG_bind_) is consist of electrostatic interaction energy (ΔE_ele_), van der Waals interaction energy (ΔE_vdw_), polar solvation free energy (ΔG_p_) and nonpolar solvation free energy (ΔG_np_)*.

**Figure 7:**
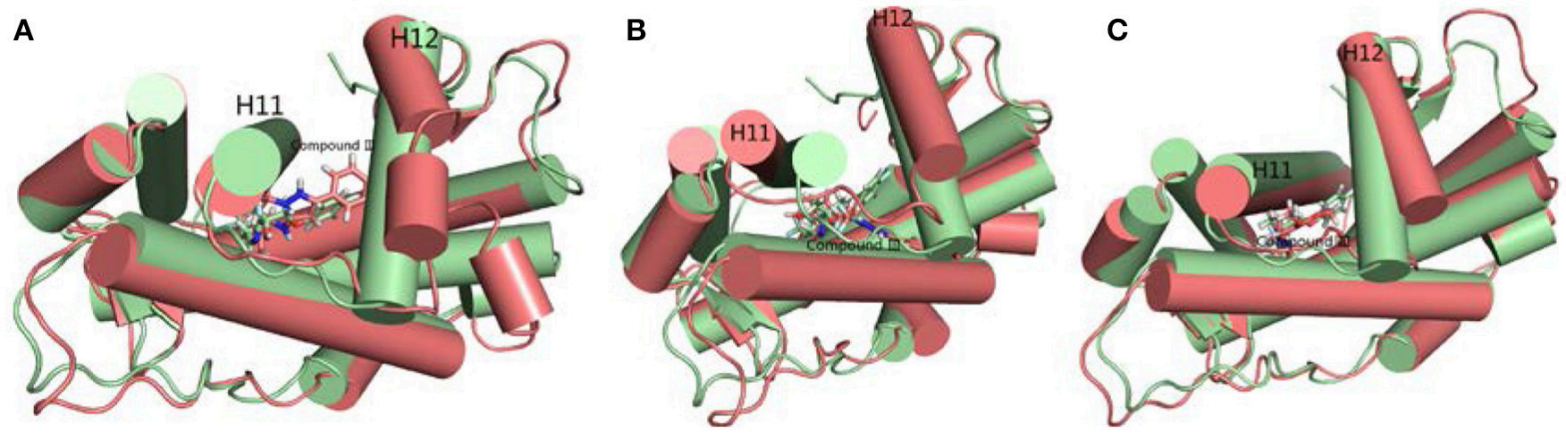
The superimposition of WT AR's initial structure with optimized structures of three systems in last 20 ns. Green cartoon and sticks represent the initial crystal structure. **(A)** The complexes of initial structure and WT (deep salmon cartoon and sticks). **(B)** The complexes of initial structure and Compound III/F876L AR (deep salmon cartoon and sticks). **(C)** The complexes of initial structure and Compound III/H874Y AR (deep salmon cartoon and sticks).

Hydrogen bond interaction (Table 6) between Compound III and ARs show that all three complexes have relatively stable hydrogen bonds during last 20 ns in simulations. For the Compound III/WT AR system, Compound III formed two hydrogen bonds with T877 (98.86%) and Q711 (51.25%), while for the Compound III/F876L AR system, Compound III formed only one stable hydrogen bond with N705 (97.94%). Two hydrogen bonds are formed between R752 (85.41%), Q711 (65.50%) and Compound III respectively. Hydrogen bonds are beneficial to stabilize the position of the benzene of Compound III to prevent H12 from closing, as shown in Figure 7.

**Table 6:** The hydrogen bonds between compound III and key residues in ARs.

**Complex**	**Donor**	**Acceptor**	Distance(Å)^*^	Angle(°)^*^	**Occupancy (%)**
Compound III/WT AR	Compound III@N3-H20	T877@OG1	2.92	156.41	98.86
	Q711@NE2-H22	Compound III@O4	3.10	153.14	51.25
Compound III/F876L AR	Compound III@N3	N705@OD1-H20	2.89	156.58	97.94
Compound III/H874YAR	R752@NH2-H22	Compound III@O2	3.04	144.22	85.41
	Q711@NE2-H22	Compound III@O1	2.83	158.61	65.50

* *The hydrogen bonds are determined by the acceptor … donor atom distance of < 0.35 nm and acceptor … H-donor angle of >120°. WT AR represents wild type AR*.

**Figure 8:**
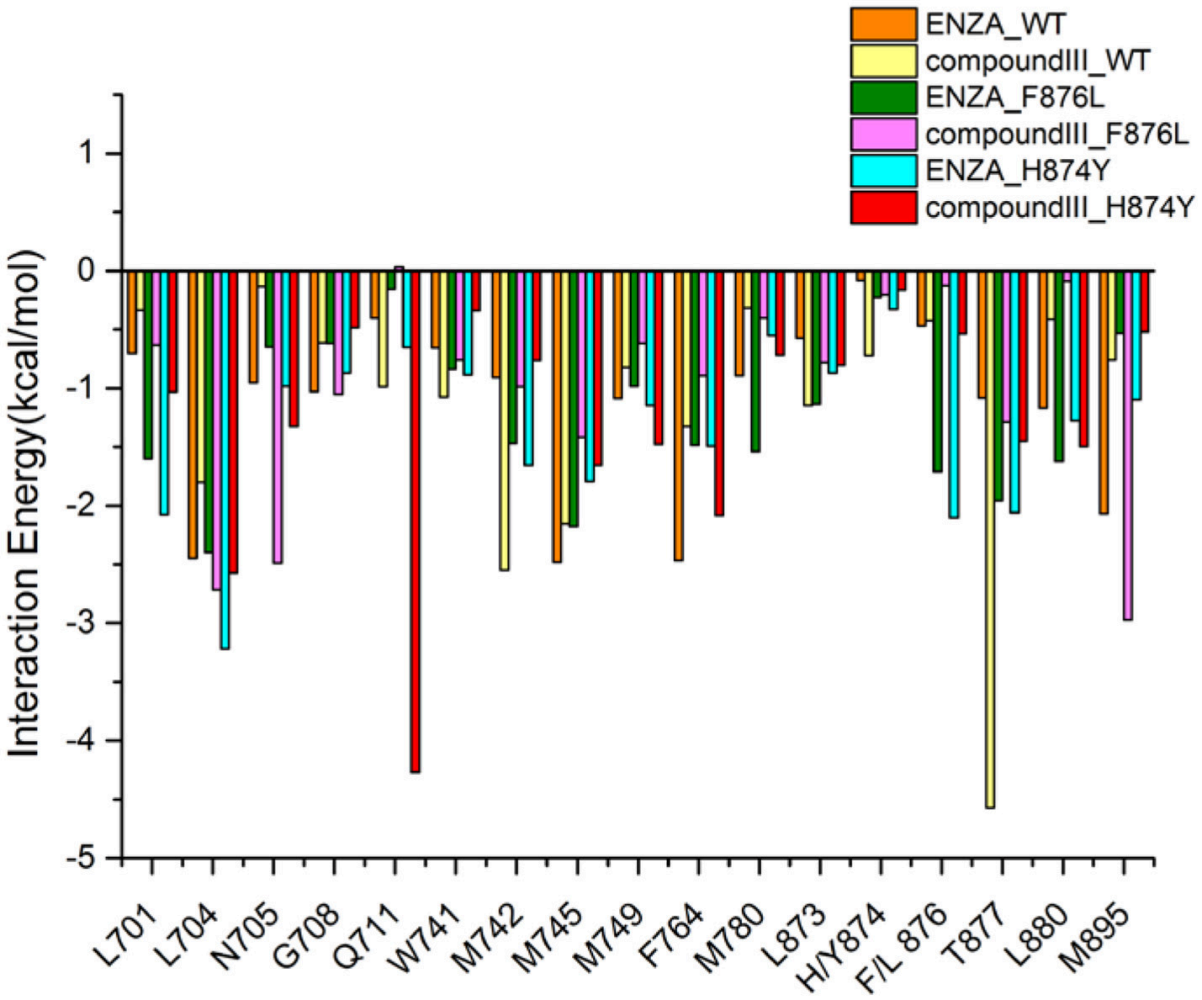
Contribution of the important residues for ligand binding. All structures are average conformations generated from the last 20 ns snapshots of each MD system.

To get the optimized structure of each system, we performed a cluster analysis for the last 20 ns trajectories using Kclust algorithm (https://mmtsb.org/workshops/sean-bin_workshop_2012/Tutorials/MMTSB_EnsembleAnalysis/MMTSBEnsembleAnalysis.html). The systems of the Compound III/WT AR, Compound III/F876L AR and Compound III/H874Y AR were clustered into 6, 7, and 7 classes and the representative conformation accounted for 44.0, 29.7, and 35.0%, respectively. From the largest number of cluster, the conformation with the lowest RMSD to the cluster centers was selected. These optimized structures were used to analyze the conformation difference between corresponding initial structure and structure after 80 ns MD simulation, shown in Figure 6. We can see the superimposition of the wild type AR and the three optimized structures respectively in Figure 7. This figure shows that for all three systems, benzene ring of Compound III is close to the H12, meanwhile, the binding free energy decomposition analysis (Figure 8) also shows that residue L/F876 has weaker interactions with enzalutamide or Compound III than enza/F876L AR and enza/H874Y AR complexes. It has been experimentally confirmed (Korpal et al., 2013) or computational predicted (Liu et al., 2017) that F876L and H874Y mutation could switch enzalutamide from AR antagonist to AR agonist. The benzene ring linked to pyrazole of Compound III is close to H12 in all these systems, pushing H12 far away from the ligand binding pocket, which distorts the AF2 site resulting the inactivation of transcription. Based on the MD simulations results above, F876L and H874Y mutations in AR cannot convert compound III from AR antagonist to AR agonist.

## Conclusion

In this study, structure-based docking and ligand-based QSAR methodology were combined to virtually screen chemical databases to discover new androgen receptor antagonists with novel chemical skeleton. After virtual screening and the cell proliferation assay validation, the inhibition rate of prostate cancer cell LNCaP and DHT-induced transcriptional activation of AR were detected on a series of pyrazolopyrimidine analogs *in vitro*. Importantly, Compound III was proved to be the one of the most potent of these non-nitrophenyl and non-cyanophenyl type nonsteroidal AR antagonists and exhibited potent antiandrogenic activity. MD simulations and MM/GBSA methods were employed to investigate the antagonist mechanism of representative Compound III to AR, which further proved that this compound can prevent H12 in AR LBD from closing to distort the formation of AF2, resulting the invalid transcription. Hence, Pyrazolopyrimidine, as a novel antiandrogenic skeleton serves as a core structure of AR antagonist, deserves to be further optimized to discover more AR antagonists with high potency.

## Materials and methods

It was reported that virtual screening strategy combining structure- and ligand-based methods improves the performance and consistency of virtual screening compared to the single methods alone (Svensson et al., 2012). In the current study, molecular docking and QSAR models were used together to search a chemical database for potential androgen receptor antagonists.

### Database preparation

Considering the diversity of the molecular structure, the commercially available ChemDiv structural library (http://www.chemdiv.com/) was used for screening, which includes 1.6 million compounds. All these molecular structures were protonated/deprotonated by generating ionization states with significant population at the specific 7.0 ± 2.0 in Maestro (Friesner et al., 2004). After adding partial charges, the molecular structures were minimized with MMFFs force field keeping its original stereochemistry.

### Structure-based virtual screening

#### Protein preparation

The androgen receptor crystal structures were downloaded from Protein Data Bank (PDB code: 1T65) and prepared by using the Protein Preparation Wizard in Maestro. The treatments on AR structure include deleting the involved solvents, adding the missing hydrogen atoms (missing loops) and residues via Prime module, protonation protein crystal structure to neutral (pH = 7), minimizing side chains using OPLS 2005 force field, and definition receptor grid using a 12 Å box centered on the ligand. All other adjustable settings were set as default. Moreover, we constructed an antagonistic AR (named Anti-AR) model according to the reported homology modeling methodology (Liu et al., 2012).

#### Docking-based virtual screening

First, all the chemical structures were collected and docked into the ligand binding domain of 1T65 and Anti-AR in Glide standard precision (SP) model, respectively. This program is generally used to approximate a systematic search of the conformational, oriented and positional space for the docked compound. The involved active site was defined from the coordination of the built-in ligand, using the default settings. The chemicals that could accommodate into the ligand binding domains of 1T65 and Anti-AR formed database 1. Then, the Lipinski's rule of five (Lipinski et al., 2001) was further utilized as a benchmark to filter the compounds to form database 2, which were submitted to the next process.

### Ligand-based virtual screening

Our group has published a predictive QSAR models (Y_1_) on androgen receptor antagonists (Wang et al., 2015), which can be used here to evaluate the *in silico* bioactivities of the screened chemicals. Furthermore, we found more AR antagonists with different structures from the literatures, and three new QSAR models (Y_2_, Y_3_, and Y_4_) were established and used in the present study. The processes to build models Y_2_, Y_3_, and Y_4_ are as follows.

#### Data set

The chemical structures together with corresponding bioactivities taken from literatures (Hamann et al., 1998; Zhi et al., 1998, 1999; Kong et al., 2000; Tachibana et al., 2007a,b; Zhao et al., 2008) were collected to develop models Y_2_ − Y_4_ respectively. The biological activities (IC_50_) were converted into negative logarithmic unit pIC_50_ and used as dependent variables. The 3D structures of these molecules were imported into SYBYL 6.9 (SYBYL, version 6.9, Tripos Inc., St. Louis, MO)^2^ to calculate Gasteiger-Hückel partial charges. The energy minimization was performed using the Tripos force field with convergence criterion of 0.01 kcal/mol·Å. Then the multi-search routine was performed to do conformation search to obtain the lowest energy conformation for each molecule. Subsequently, the compounds were randomly split into training sets and prediction sets.

#### Descriptors generation

The obtained lowest energy confirmations were submitted to DRAGON 5.5 (DRAGON for Windows, version 5.5)^3^ to calculate all the 20 types of descriptors. To decrease redundant information, the calculated descriptors were pretreated to exclude the constant or near constant value (variance < 1) and high correlated pairwise (*R* > 0.99).

#### Modeling method and validation

Here in this study, genetic algorithm was employed to select important descriptors highly related to the bioactivities, using the MLR method to build the QSAR models, executed in QSARINS (Gramatica et al., 2013). To thoroughly validate the built QSAR models, several internal and external validation standards were combined to evaluate the robustness and predictive ability of the built models (Gramatica and Sangion, 2016). After strict validation, these models were used to predict the bioactivities of the screened chemicals. The chemicals with high *in silico* bioactivities formed database 3.

### Structural analysis

After the structure- and ligand-based virtual screening, chemicals with high binding affinities and high *in silico* bioactivities were filtered out for the succeeding analysis. Molecules with similar skeleton were extracted.

Then, according to our experience on medicinal chemistry and combining with the docking and QSAR results, the novel skeleton of pyrazolopyrimidine was eventually proposed for further research. To thoroughly explore the pyrazolopyrimidine analogs, the specific skeleton was used as a substructure to search the ZINC online database (http://zinc15.docking.org/), and the resulted chemicals were downloaded to form a new pyrazolopyrimidine database 4. Subsequently, LigandFit and Libdock modules were used to dock all these pyrazolopyrimidine analogs to the ligand binding domain of 1T65 and Anti-AR protein, and the four QSAR models were employed to predict corresponding bioactivities. At the end, the hit chemicals with excellent performance both in the docking and QSAR model predictions were selected for further experimental validation.

### *In vitro* assay and biological evaluation

#### Materials preparation

The selected chemicals were purchased from the established suppliers, including the J&K Chemicals and SPECS.

#### Cell proliferation assay

The LNCaP cells were cultured in RPMI 1640 medium containing 10% FBS, 100 unit/ml penicillin and 100 unit/ml streptomycin at 37°C in a humidified atmosphere with 5% CO_2_. Before being treated with compounds, cells were cultured in 100 mm cell dish at a density of 5 × 10^5^ per well, then sub-cultured when it grew to approximate 90% confluence. Subsequently, the compounds were evaluated in a cellular assay by measuring IC_50_ values. All experiments were performed during the logarithmic phase of cell growth. For analysis of the effect of all chemicals on LNCaP cell proliferation, the cells were seeded in 96 well plates at a density of 5,000 LNCaP cells per well and treated with chemicals for 120 h. A 20 μL aliquot of tetrazole (MTT) was added to each well. After 4 h incubation, 150 μL of isopropanol was added to dissolve the crystal. Finally, the absorbance was measured at a wavelength 570 nm.

#### AR reporter gene assay

Luciferase reporter assay based on androgen response elements (ARE) was used to examine chemicals for androgenic activity. COS-7 cells were seeded in a 24 well plate, and cultured in medium (DMEM medium containing 10% charcoal-stripped FBS (CSS), 2 mM glutamine) for 24 h in a 24 well plate. The plasmids of pcDNA3.1-AR, pMMTV-Luc and pRL-SV40 were transfected in COS-7 cells by using transfection reagent Lipofectamine 3000 according to the instructions of manufacturer. After culturing at 37°C in a 5% CO_2_ atmosphere for 24 h, cells were treated with the chemicals (final concentration, 10 μM) in the presence of 10 nM DHT for 24 h. Then, cells were harvested with 150 μL of cell passive lysis buffer (Promega). The firefly and renilla luciferase activities were determined with a Dual-Glo Luciferase Assay Kit (Promega). The data were obtained in triplicate and expressed as inhibition rate over the DHT control. Inhibition% = 1−(RLU_test_ − RLU_blank_)/(RLU_DHT_ − RLU_blank_) × 100%. RLU = relative light unit.

### Molecular dynamics simulations

The crystal structures of WT, H874Y mutant AR (PDB ID 2Q7L) were obtained from the Protein Data Bank (http://www.rcsb.org/pdb)^4^. Then Pymol (v1.7) program was used to generate the 3D structure of F876L mutant AR by mutated wild type AR 876F to 876L. Then the CDOCKER module of Discovery Studio 2.5 [(Discovery Studio version 2.5, 2009) Accelrys Inc. CA] was used to dock Compound III to all the three kinds of ARs. The lowest-energy models were selected as the docking results.

MD simulations were performed with AMBER12 (Case et al., 2012) package. The geometry optimization and partial charges calculation of small-molecule ligand were performed in Gaussian09 program using HF/6-31G^*^(Frisch et al., 2009) basis set. Then the restrained electrostatic protential (RESP) (Bayly et al., 1993; Cieplak et al., 1995; Fox and Kollman, 1998) of Compound III was assigned using the general AMBER force filed (GAFF) (Wang et al., 2004). The ff99SB force field (Hornak et al., 2006) was used to parameterize the systems. Finally, all the systems were neutralized with chloride ions and immersed in a cubic TIP3P water box (Ryckaert et al., 1977). The solvent boundary was at least 10Å away from the proteins. Subsequently, all systems were minimized using a steepest decent method followed by conjugate gradient method, and then heated from 0 to 310 K. The temperature was controlled by Langevin thermostat with a coupling coefficient of 2.0 ps^−1^. SHAKE algorithm (Eaamann et al., 1995) was used to constrained bond lengths involving hydrogen atoms. All equilibration and subsequent MD stages were carried out in the isothermal isobaric (NTP) ensemble with a target pressure of 1 bar. The time step was set as 2 fs and the production time for all the systems was 100 ns.

The binding free energy of each system was calculated using molecular mechanics/generalized born surface area (MM/GBSA) methodology (Massova and Kollman, 2000; Xu et al., 2013; Sun et al., 2014a,b). The binding free energy (ΔG_bind_) is consisted of electrostatic interaction energy (ΔE_ele_), van der Waals interaction energy (ΔE_vdw_), polar solvation free energy (ΔG_p_) and nonpolar solvation free energy (ΔG_np_). Dielectric constants of 1.0 and 80.0 were set for solute and solvent respectively. The polar part of desolvation (ΔG_GB_) was computed with the modified GB model developed by Onufriev et al. (referred to as igb = 2 in Amber) (Onufriev et al., 2004), and the nonpolar part of desolvation (ΔG_SA_) was determined based on the solvent accessible surface area (SASA) computed by the LCPO algorithm: (Weiser et al., 1999) ΔG_SA_ = 0.0072ΔSASA. The change of the conformational entropy (−TΔS) was not considered due to high computational cost and low prediction accuracy (Wang et al., 2006; Hou et al., 2011). In total, 2,000 snapshots evenly extracted from 80 to 100 ns were used to calculate the energy terms. For each systems, the interaction spectrum between Compound III and ARs on a per-residue basis was calculated by MM/GBSA decomposition analysis supported by the mmpbsa.py module in AMBER (Gohlke et al., 2003; Hou et al., 2008, 2012).

## Author contributions

XW and JL conceived and coordinated the study. LW did the structure-based virtual screening, screening of pyrazolopyrimidine analogs, *in vitro* evaluation of hit compounds, TS did the molecular docking, molecular dynamics simulations, and they wrote the paper. All authors analyzed the results and approved the final version of the manuscript.

### Conflict of interest statement

The authors declare that the research was conducted in the absence of any commercial or financial relationships that could be construed as a potential conflict of interest.

## Footnotes

^1^DRAGON for Windows(Software for Molecular Descriptor Calculations), version 5.5 - Talet srl.http://www.talete.mi.it/

^2^SYBYL version 6.9. Tripos Inc., St. Louis, M. O.

^3^DRAGON for Windows(Software for Molecular Descriptor Calculations), version 5.5 - Talet srl. http://www.talete.mi.it/

^4^RCSB Protein Data Bank. http://www.rcsb.org/pdb

## Supplementary material

Molecular structures and corresponding biological activities used in this study to build QSAR models Y2-Y4 were listed. Definitions of external validation metrics were listed in Table S4. The predicted biological activity from the Y1-Y4 models and the docking affinities were listed in Table S5.
