# Supplementary material for: Discovery and Identification of Pyrazolopyramidine Analogs as Novel Potent Androgen Receptor Antagonists
Source: Front Pharmacol. 2018 Aug 28;9:864. doi: 10.3389/fphar.2018.00864 (PMC6121070; doi:10.3389/fphar.2018.00864)
Supplement: Supplementary file 1 [file Table_1.DOCX]

Table S1. The structures, experimental/predicted antiandrogenic activities and applied descriptors in the second Y2 model.

| No | Structure | Exp | Pred | Descriptors | | | |
| --- | --- | --- | --- | --- | --- | --- | --- |
|  |  |  |  | IC5 | GATS5e | DISPp | HATS3u |
| 1 ^a^ |  | 6.04 | 5.91 | 5.75 | 0.64 | 0.14 | 0.30 |
| 2 ^a^ |  | 6.14 | 6.15 | 5.70 | 0.63 | 0.28 | 0.31 |
| 3 ^a^ |  | 5.70 | 5.81 | 5.75 | 0.63 | 0.35 | 0.28 |
| 4 |  | 6.77 | 6.88 | 5.77 | 0.58 | 0.28 | 0.27 |
| 5 |  | 6.18 | 6.23 | 5.82 | 0.44 | 0.21 | 0.27 |
| 6 ^a^ |  | 6.52 | 6.71 | 5.87 | 0.67 | 0.13 | 0.26 |
| 7 |  | 5.24 | 6.76 | 5.91 | 0.43 | 0.32 | 0.26 |
| 8 |  | 6.07 | 5.95 | 5.87 | 0.44 | 0.26 | 0.26 |
| 9 |  | 6.60 | 6.53 | 5.91 | 0.67 | 0.34 | 0.27 |
| 10 |  | 6.38 | 6.27 | 5.96 | 0.67 | 0.43 | 0.25 |
| 11 |  | 6.04 | 6.06 | 5.96 | 0.44 | 0.15 | 0.25 |
| 12 ^a^ |  | 6.48 | 6.48 | 6.00 | 0.66 | 0.13 | 0.26 |
| 13 |  | 6.28 | 5.80 | 6.04 | 0.66 | 0.27 | 0.24 |
| 14 |  | 5.54 | 5.47 | 5.94 | 0.52 | 0.27 | 0.28 |
| 15 |  | 6.47 | 6.17 | 5.90 | 0.79 | 0.45 | 0.27 |
| 16 |  | 5.47 | 5.81 | 5.95 | 0.80 | 0.18 | 0.26 |
| 17 ^a^ |  | 5.74 | 5.51 | 5.95 | 0.81 | 0.43 | 0.25 |
| 18 |  | 5.89 | 5.80 | 5.94 | 0.81 | 0.50 | 0.25 |
| 19 |  | 5.46 | 5.61 | 5.90 | 0.82 | 0.23 | 0.24 |
| 20 |  | 5.96 | 6.35 | 5.86 | 0.80 | 0.41 | 0.29 |
| 21 ^a^ |  | 6.38 | 6.12 | 5.76 | 0.71 | 0.15 | 0.26 |
| 22 |  | 6.38 | 6.07 | 5.81 | 0.70 | 0.36 | 0.29 |
| 23 |  | 6.72 | 6.64 | 5.66 | 0.70 | 0.34 | 0.28 |
| 24 |  | 6.52 | 6.46 | 5.70 | 0.71 | 0.39 | 0.28 |
| 25 |  | 5.80 | 5.84 | 5.93 | 0.92 | 0.41 | 0.27 |
| 26 |  | 6.14 | 6.30 | 5.76 | 0.64 | 0.08 | 0.27 |
| 27 |  | 6.52 | 6.46 | 5.73 | 0.71 | 0.16 | 0.30 |
| 28 |  | 6.31 | 6.48 | 5.79 | 0.81 | 0.22 | 0.31 |
| 29 |  | 6.35 | 6.18 | 5.70 | 0.69 | 0.31 | 0.31 |
| 30 |  | 6.32 | 6.06 | 5.74 | 0.69 | 0.31 | 0.29 |
| 31 |  | 6.03 | 5.80 | 5.79 | 0.70 | 0.37 | 0.31 |
| 32 |  | 5.74 | 5.60 | 5.84 | 0.80 | 0.40 | 0.29 |
| 33 |  | 5.20 | 5.50 | 5.89 | 0.80 | 0.38 | 0.29 |
| 34 |  | 5.18 | 5.24 | 5.98 | 0.84 | 0.44 | 0.28 |
| 35 |  | 6.70 | 6.59 | 5.61 | 0.69 | 0.12 | 0.30 |
| 36 |  | 6.28 | 6.59 | 5.64 | 0.70 | 0.40 | 0.29 |

^a^ the prediction set samples.
