# Supplementary material for: Discovery and Identification of Pyrazolopyramidine Analogs as Novel Potent Androgen Receptor Antagonists
Source: Front Pharmacol. 2018 Aug 28;9:864. doi: 10.3389/fphar.2018.00864 (PMC6121070; doi:10.3389/fphar.2018.00864)
Supplement: Supplementary file 2 [file Table_2.DOCX]

Table S2. The structures, experimental/predicted antiandrogenic activities and applied descriptors in the third Y3 model.

| No | Structure | Exp | Pred | Descriptors | | |
| --- | --- | --- | --- | --- | --- | --- |
|  |  |  |  | IVDE | C-009 | BLTF96 |
| 1 |  | 8.06 | 7.67 | 1.73 | 0.00 | -4.56 |
| 2 |  | 7.23 | 7.17 | 1.44 | 0.00 | -3.53 |
| 3 |  | 7.59 | 7.43 | 1.44 | 0.00 | -3.30 |
| 4 ^a^ |  | 5.80 | 6.73 | 1.47 | 0.00 | -4.11 |
| 5 |  | 8.52 | 8.16 | 1.74 | 0.00 | -4.15 |
| 6 |  | 5.82 | 5.71 | 1.62 | 0.00 | -5.77 |
| 7 |  | 5.59 | 5.77 | 1.66 | 0.00 | -5.94 |
| 8 |  | 6.00 | 6.55 | 1.50 | 0.00 | -4.39 |
| 9 ^a^ |  | 6.21 | 7.16 | 1.49 | 0.00 | -3.82 |
| 10 ^a^ |  | 7.55 | 7.16 | 1.49 | 0.00 | -3.82 |
| 11 |  | 8.40 | 7.77 | 1.52 | 0.00 | -3.39 |
| 12 |  | 8.00 | 7.90 | 1.72 | 0.00 | -4.32 |
| 13 |  | 7.21 | 7.32 | 1.51 | 0.00 | -3.78 |
| 14 |  | 7.68 | 7.21 | 1.53 | 0.00 | -3.97 |
| 15 ^a^ |  | 8.30 | 7.96 | 1.49 | 0.00 | -3.09 |
| 16 |  | 5.89 | 5.89 | 1.49 | 1.00 | -3.43 |
| 17 |  | 5.63 | 5.62 | 1.48 | 1.00 | -3.63 |
| 18 |  | 6.60 | 6.92 | 1.49 | 0.00 | -4.03 |
| 19 ^a^ |  | 7.96 | 8.13 | 1.71 | 0.00 | -4.05 |
| 20 |  | 8.89 | 8.42 | 1.72 | 0.00 | -3.86 |
| 21 |  | 7.30 | 7.92 | 1.73 | 0.00 | -4.34 |
| 22 |  | 8.10 | 7.71 | 1.69 | 0.00 | -4.32 |
| 23 |  | 7.57 | 8.13 | 1.71 | 0.00 | -4.05 |
| 24 ^a^ |  | 5.73 | 5.58 | 1.55 | 0.00 | -5.53 |
| 25 |  | 5.57 | 5.53 | 1.62 | 0.00 | -5.93 |
| 26 |  | 5.67 | 5.43 | 1.62 | 0.00 | -6.02 |
| 27 ^a^ |  | 5.63 | 5.77 | 1.62 | 0.00 | -5.71 |
| 28 |  | 6.10 | 6.08 | 1.77 | 0.00 | -6.19 |
| 29 |  | 5.23 | 5.52 | 1.55 | 0.00 | -5.59 |
| 30 |  | 5.84 | 5.61 | 1.55 | 0.00 | -5.51 |
| 31 |  | 5.73 | 5.59 | 1.66 | 0.00 | -6.10 |
| 32 |  | 5.80 | 5.68 | 1.66 | 0.00 | -6.02 |
| 33 |  | 5.46 | 5.49 | 1.66 | 0.00 | -6.19 |

^a^ the prediction set samples.
