# Supplementary material for: Discovery and Identification of Pyrazolopyramidine Analogs as Novel Potent Androgen Receptor Antagonists
Source: Front Pharmacol. 2018 Aug 28;9:864. doi: 10.3389/fphar.2018.00864 (PMC6121070; doi:10.3389/fphar.2018.00864)
Supplement: Supplementary file 3 [file Table_3.DOCX]

Table S3. The structures, experimental/predicted antiandrogenic activities and applied descriptors in the forth Y4 model.

| No | Structure | Exp | Pred | Descriptors | | | | |
| --- | --- | --- | --- | --- | --- | --- | --- | --- |
|  |  |  |  | IC1 | F05[N-F] | R4u | Depressant-80 | HATS7m |
| 1 |  | 7.57 | 6.24 | 3.48 | 3.00 | 2.10 | 0.00 | 0.15 |
| 2 |  | 7.52 | 6.34 | 3.48 | 3.00 | 2.10 | 0.00 | 0.18 |
| 3 |  | 6.17 | 7.41 | 3.74 | 3.00 | 2.15 | 1.00 | 0.12 |
| 4 ^a^ |  | 7.52 | 5.43 | 3.71 | 6.00 | 2.07 | 0.00 | 0.13 |
| 5 |  | 7.51 | 6.56 | 3.79 | 6.00 | 1.90 | 1.00 | 0.31 |
| 6 |  | 5.84 | 7.62 | 3.76 | 3.00 | 2.24 | 1.00 | 0.12 |
| 7 |  | 7.66 | 7.62 | 3.76 | 3.00 | 2.24 | 1.00 | 0.12 |
| 8 |  | 6.11 | 7.38 | 3.84 | 3.00 | 1.83 | 1.00 | 0.15 |
| 9 ^a^ |  | 7.14 | 7.37 | 3.66 | 3.00 | 2.21 | 1.00 | 0.15 |
| 10 |  | 6.61 | 7.36 | 3.84 | 3.00 | 1.85 | 1.00 | 0.14 |
| 11 |  | 6.67 | 6.22 | 3.48 | 3.00 | 2.11 | 0.00 | 0.15 |
| 12 |  | 8.05 | 7.33 | 3.66 | 3.00 | 2.23 | 1.00 | 0.13 |
| 13 ^a^ |  | 6.12 | 7.56 | 3.66 | 3.00 | 2.21 | 1.00 | 0.20 |
| 14 |  | 5.73 | 6.40 | 3.42 | 3.00 | 2.12 | 1.00 | 0.14 |
| 15 |  | 6.05 | 6.99 | 3.57 | 3.00 | 2.25 | 1.00 | 0.12 |
| 16 |  | 7.20 | 6.19 | 3.58 | 3.00 | 1.94 | 0.00 | 0.12 |
| 17 |  | 6.47 | 5.80 | 3.37 | 3.00 | 2.10 | 0.00 | 0.14 |
| 18 |  | 6.32 | 6.05 | 3.50 | 3.00 | 1.91 | 0.00 | 0.17 |
| 19 |  | 5.49 | 6.91 | 3.66 | 3.00 | 1.99 | 1.00 | 0.12 |
| 20 ^a^ |  | 5.94 | 6.07 | 3.44 | 3.00 | 2.16 | 0.00 | 0.12 |
| 21 |  | 6.30 | 6.86 | 3.69 | 3.00 | 1.90 | 1.00 | 0.11 |
| 22 ^a^ |  | 6.20 | 5.81 | 3.69 | 6.00 | 2.16 | 1.00 | 0.10 |
| 23 |  | 6.33 | 6.17 | 3.69 | 6.00 | 1.84 | 0.00 | 0.42 |
| 24 |  | 6.47 | 6.30 | 3.56 | 3.00 | 2.08 | 0.00 | 0.12 |
| 25 |  | 6.60 | 6.68 | 3.68 | 3.00 | 1.87 | 0.00 | 0.19 |
| 26 |  | 6.76 | 6.57 | 3.50 | 3.00 | 2.24 | 0.00 | 0.17 |
| 27 |  | 7.14 | 6.88 | 3.50 | 3.00 | 2.26 | 1.00 | 0.14 |
| 28 |  | 6.14 | 6.90 | 3.50 | 3.00 | 2.24 | 0.00 | 0.26 |
| 29 |  | 7.35 | 6.51 | 3.42 | 3.00 | 2.27 | 1.00 | 0.11 |
| 30 |  | 7.57 | 6.96 | 3.58 | 3.00 | 2.19 | 1.00 | 0.12 |
| 31 ^a^ |  | 7.44 | 7.29 | 3.45 | 0.00 | 1.89 | 1.00 | 0.07 |
| 32 |  | 7.57 | 7.62 | 3.76 | 3.00 | 2.24 | 1.00 | 0.12 |
| 33 |  | 7.24 | 7.31 | 3.80 | 3.00 | 1.97 | 1.00 | 0.12 |

^a^ the prediction set samples.
