# Supplementary material for: Discovery and Identification of Pyrazolopyramidine Analogs as Novel Potent Androgen Receptor Antagonists
Source: Front Pharmacol. 2018 Aug 28;9:864. doi: 10.3389/fphar.2018.00864 (PMC6121070; doi:10.3389/fphar.2018.00864)
Supplement: Supplementary file 4 [file Table_4.docx]

Table S4. Definitions of external validation metrics

| Metric | Reference |
| --- | --- |
| $Q_{F1}^{2}=1-\frac{\sum_{i=1}^{n_{EXT}} \left( \hat{y}_{i}-y_{i} \right)^{2}}{\sum_{i=1}^{n_{EXT}} \left( y_{i}-\left\langle y_{TR} \right\rangle\right)^{2}}$ | Tropsha et al.^1^ |
| $Q_{F2}^{2}=1-\frac{\sum_{i=1}^{n_{EXT}} \left( \hat{y}_{i}-y_{i} \right)^{2}}{\sum_{i=1}^{n_{EXT}} \left( y_{i}-\left\langle y_{EXT} \right\rangle\right)^{2}}$ | Schüürmann et al.^2^ |
| $Q_{F3}^{2}=1-\frac{\left[ \sum_{i=1}^{n_{EXT}} \left( \hat{y}_{i}-y_{i} \right)^{2} \right]/{n_{EXT}}}{\left[ \sum_{i=1}^{n_{EXT}} \left( y_{i}-\left\langle y_{TR} \right\rangle\right)^{2} \right]/{n_{TR}}}$ | Consonni et al.^3^ |
| $CCC=\frac{2\sum_{i=1}^{n} \left( y_{i}-\left\langle y \right\rangle\right)\left( \hat{y}_{i}-\left\langle\hat{y} \right\rangle\right)}{\sum_{i=1}^{n} \left( y_{i}-\left\langle y \right\rangle\right)^{2}+\sum_{i=1}^{n} \left( \hat{y}_{i}-\left\langle\hat{y} \right\rangle\right)+n\left( \left\langle y \right\rangle-\left\langle\hat{y} \right\rangle\right)^{2}}$ | Chirico et al. ^4^ |

*n* is the number of molecules in the whole dataset; *n_TR_* is the number of molecules in the training set; *n_EXT_* is the number of molecules in the valiation set; $\hat{y}_{i}$ is the calculated property for the molecule i; $y_{i}$ is the experimental property for the molecule i; $\left\langle y \right\rangle$ is the average of experimental properties for the whole dataset; $\left\langle\hat{y} \right\rangle$ is the average of calculated properties for the whole dataset; $\left\langle y_{TR} \right\rangle$ is the average of experimental properties for the training set; $\left\langle y_{EXT} \right\rangle$ is the average of experimental properties for the validation set.

**References**

1. Tropsha, A., Gramatica, P., and Gombar, V. K. (2003). The importance of being earnest: Validation is the absolute essential for successful application and interpretation of QSPR models. *J. Comb.Sci.* **22**, 69.

2. Schüürmann, G., Ebert, R. U., Chen, J., Wang, B., and Kühne, R. (2008). External validation and prediction employing the predictive squared correlation coefficient test set activity mean vs training set activity mean. *J. Chem. Inf. Model.* **48**, 2140-2145. doi: 10.1021/ci800253u

3. Consonni, V., Ballabio, D., and Todeschini, R. (2009). Comments on the definition of the Q2 parameter for QSAR validation.*J. Chem. Inf. Model.* **49**, 1669-1678. doi: 10.1021/ci900115y

4. Chirico, N., andGramatica, P. (2001). Real external predictivity of QSAR models: how to evaluate it? Comparison of different validation criteria and proposal of using the concordance correlation coefficient.*J. Chem. Inf. Model.* **51**, 2320-2335. doi: 10.1021/ci200211n
