# Supplementary material for: Discovery and Identification of Pyrazolopyramidine Analogs as Novel Potent Androgen Receptor Antagonists
Source: Front Pharmacol. 2018 Aug 28;9:864. doi: 10.3389/fphar.2018.00864 (PMC6121070; doi:10.3389/fphar.2018.00864)
Supplement: Supplementary file 5 [file Table_5.docx]

Table S5. The predicted biological activity from the Y_1_-Y_4_ models and the docking affinities.

| No | Predicted bioactivity | | | | LibDock Score | | LigandFit Score | |
| --- | --- | --- | --- | --- | --- | --- | --- | --- |
|  | Y_1_ | Y_2_ | Y_3_ | Y_4_ | 1T65 | Anti-AR | 1T65 | Anti-AR |
| CompoundⅠ | 7.548 | 9.396 | 9.838 | 7.636 | 94.6553 | 87.876 | 46.195 | 49.537 |
| CompoundⅡ | 7.608 | 7.404 | 8.371 | 9.074 | 97.8519 | 104.094 | 35.297 | 52.455 |
| CompoundⅢ | 7.720 | 8.494 | 7.907 | 6.497 | 104.651 | 103.916 | 64.369 | 49.976 |
| CompoundⅣ | 7.345 | 8.992 | 9.004 | 7.673 | 105.059 | 123.321 | 8.359 | 48.492 |
| R-Bicalutamide | 7.541 | 7.995 | 9.353 | 6.037 | 117.796 | 122.574 | 18.435 | 58.888 |
